# Diagnosis of celiac disease and applicability of ESPGHAN guidelines in Mediterranean countries: a *real life* prospective study

**DOI:** 10.1186/s12876-017-0577-x

**Published:** 2017-01-21

**Authors:** Andrea Smarrazzo, Zrinjka Misak, Stefano Costa, Dušanka Mičetić-Turk, Mona Abu-Zekry, Aydan Kansu, Abdelhak Abkari, Karim Bouziane-Nedjadi, Mongi Ben Hariz, Eleftheria Roma, Virtut Velmishi, Maria Legarda Tamara, Thomas Attard, Veselinka Djurisic, Luigi Greco, Giuseppe Magazzù

**Affiliations:** 1 Department of Translational Medical Sciences, School of Medicine, University “Federico II”, Naples, Italy; 2 European Laboratory for Food Induced Diseases, Naples, Italy; 3 Children’s Hospital Zagreb, Zagreb, Croatia; 4 Celiac Regional Centre, Pediatric Gastroenterology and Cystic Fibrosis Unit, University of Messina, Via Consolare Valeria 1, 98125, Messina, Italy; 5 Paediatric Department, University Medical Centre, Maribor, Slovenia; 6 Cairo University Children Hospital, El Cairo, Egypt; 7 Department of Pediatric Gastroenterology, School of Medicine, Ankara University, Ankara, Turkey; 8 Centre Hospitalier Universitaire Ibnou Rochd, Casablanca, Morocco; 9 Faculté de Médecine d’Oran, Oran, Algeria; 10 Pediatric Unit, Mongi SLIM’s Hospital of Tunis, Marsa, Tunisia; 11 First Department of Pediatrics, University of Athens, Athens, Greece; 12 Service of Pediatric Gastroenterology “Mother Teresa” Hospital, Tirana, Albania; 13 Paediatric Gastroenterology Unit, Cruces University Hospital, Bilbao, Spain; 14 University of Malta, Msida, Malta; 15 Clinical Center of Montenegro, Podgorica, Montenegro

**Keywords:** Celiac disease, Diagnosis, Mediterranean area, ESPGHAN

## Abstract

**Background:**

We assessed how the diagnosis of Celiac Disease (CD) is made and how the new ESPGHAN guidelines can be applied in children from countries with different resources.

**Methods:**

A *real life* prospective study was performed in 14 centres of 13 different Mediterranean countries. Participants were asked to apply the usual diagnostic work-up for CD according to their diagnostic facilities.

**Results:**

There were 1974 patients enrolled in the study, mean age 4 years, 10 months; 865 male, 1109 female. CD was confirmed in 511 (25.9%) and was unconfirmed in 1391 (70.5%) patients; 14 patients were diagnosed as having CD according to the new ESPGHAN guidelines, 43 patients were classified as having potential CD. In all participating countries the diagnosis of CD relied on histology of duodenal biopsy; in 5 countries, HLA, and in one country endomysial antibodies (EMA) were not available. Symptoms did not add a significant increase to the pre-test probability of serological tests. The positive predictive value of tissue transglutaminase type 2 (tTG) antibodies performed with different kits but all corresponding to those recommended by ESPGHAN was 96.1% (95% CI 94–97.9%) in presence of tTG > 10xULN. In 135 patients with tTG >10xULN, HLA genotyping was performed and in all it was compatible with CD.

**Conclusions:**

The results of our study show that CD diagnosis still relies on intestinal biopsy in the Mediterranean area. New ESPGHAN criteria are not applicable in 5 countries due to lack of resources needed to perform HLA genotyping and, in one country, EMA assay. Further simplification of the new ESPGHAN guidelines might be made according to what preliminarily the present results suggest if confirmed by new prospective studies.

## Background

Prevalence of celiac disease (CD) has been estimated at around 1% in Western populations [1] but most patients remain undiagnosed. The burden of unrecognized CD in countries with poor resources and facilities for diagnosis is very heavy [2]. Factors influencing the onset of this non communicable epidemics have been taken into consideration also recently [3–8]. New ESPGHAN guidelines [9] state that the protocol for the diagnosis of CD changed as a result of the availability of CD-specific tissue transglutaminase type 2 antibodies (tTG). As in children and adolescents with signs or symptoms suggestive of CD and high tTG titers with levels >10 times Upper Limits of Normal (ULN), the likelihood of villous atrophy (Marsh type 3) is very high, it has been suggested [9] that histological assessment may be omitted in symptomatic patients in whom these high tTG levels are verified by endomysial antibodies (EMA) positivity and are HLA-DQ2 and/or HLA-DQ8 heterodimer positive.

However ESPGHAN states that it is necessary to perform prospective research studies.

After the publication of the new ESPGHAN criteria, apart from a large study investigating antibody diagnostics in paediatric CD [10], 2 prospective studies [11, 12] have been performed to our knowledge. One other prospective study adopted criteria other than those suggested by the guidelines [13].

Two main problems should be highlighted:

- there are many different tTG antibody tests;
- new ESPGHAN criteria were applied in some studies, but these studies have been performed, with only one exception [12], in tertiary centres of affluent countries in subjects with classical gastrointestinal disease.

Therefore, the conclusions of a recent commentary on the applicability of the new ESPGHAN Guidelines for diagnosing CD in children from resource limited countries [14] rely on a single centre prospective study comprising only 142 children.

The Mediterranean Network for the Management of Food-Induced Diseases (MEDICEL) is a EUROMED-based action in which Mediterranean countries with different resources and diagnostic facilities participate. It, therefore, represents the *real life* ideal setting in which the new ESPGHAN guidelines can be prospectively applied.

The objectives of this prospective study were to assess how the diagnosis of CD is made in different countries and how the new ESPGHAN guidelines can be applied in different Mediterranean countries.

## Methods

### Study design

A *real life* prospective study was performed in 14 centres of 13 different Mediterranean countries participating to the MEDICEL network; all unselected new cases referred to these Centres for suspected CD and asymptomatic subjects with autoimmune CD-associated diseases or familiarity for CD were enrolled, from April 2013 to July 2014.

Participants were asked to apply the usual diagnostic work-up for CD according to their diagnostic facilities and to classify enrolled subjects as confirmed or unconfirmed CD according to shared criteria, as done in their usual clinical practice. Two sessions of shared agreement on diagnostic criteria were run through the MEDICEL network before starting the study.

Criteria for admission were: age below 18 years, clinical signs and symptoms of CD (systemic, gastrointestinal, extraintestinal) and/or associated autoimmune diseases (type 1 diabetes mellitus, thyroiditis, other autoimmune diseases) and/or no symptoms but familiarity for CD (1st and 2nd degree).

Criteria for exclusion were: already known diagnoses of CD only.

Familiarity, associated diseases, clinical symptoms, tTG as N x Upper Limit Normal (ULN), EMA, histology (Marsh-Oberhuber classification) [15], were collected into the database.

HLA-DQ2/DQ8 and follow-up were performed to confirm uncertain cases.

### Diagnostic procedures

The tTG, EMA and HLA typing methods utilized by the Centres participating in the study, if available, are shown in Table 1.

**Table 1:** tTG, EMA and HLA typing methods utilized by the Centres participating in the study

Country	tTG kit	EMA substrate	HLA typing kit
Albania	Orgentec	Monkey esophagus	NA
Algeria	Phadia - EliA Celikey	Monkey esophagus	NA
Croatia	Phadia - EliA Celikey	Monkey esophagus	Tepnel LIfecodes Corporation
Egypt	Euroimmun	NA	NA
Greece	Inova	Monkey esophagus	Olerup, HLA typing kits
Italy (ME)	Euroimmun	Umbilical cord	BioDiagene - DQ-CD Typing Plus kit
Italy (NA)	Eurospital	Monkey esophagus	BioDiagene - DQ-CD Typing Plus kit
Malta	Orgentec	Monkey esophagus	Invitrogen / Life technologies
Montenegro	Aesku	Monkey esophagus	Olerup, HLA typing kits
Morocco	Orgentec	Monkey esophagus	NA
Slovenia	Eurospital	Monkey esophagus	Olerup, HLA typing kits
Spain	Celikey; Pharmacia & Upjohn	Monkey esophagus	Tepnel LIfecodes Corporation
Tunisia	Inova	Monkey esophagus	NA
Turkey	Orgentec	Monkey esophagus	Olerup, HLA typing kits

tTG were determined with a kit of the 14 most frequently applied serum anti-TG2 IgA antibody assays taken in consideration in the new ESPGHAN guidelines (9).

With the exception of 1 centre, that utilized umbilical cord as substrate, all Centres assayed EMA on monkey esophagus as substrate.

In each Centre at least 4 endoscopic biopsy samples from duodenum, including one from bulbus, were taken.

Data provided by participants to the study were re-evaluated by A.S., L.G. and G.M, and classified as shown in Table 2.

**Table 2:** Classification of the patients’ diagnoses

Classification	Histology	tTG	EMA	HLA
CCD^a^	≥ Marsh type 2	Positive or not done	Positive or not done	Positive or not done
NCCD	Not done	>10 x ULN	Positive	Positive
UCD	Not done or Marsh type 0-1	<5 x ULN	Negative	Not done
PCD	Marsh 0-1	Positive	Positive	Positive
High probability CD^b^	Not done	>10 x ULN	Positive or not done	Positive or not done

*CD* Celiac Disease, *CCD* Confirmed Celiac Disease, *NCCD* New Criteria Celiac Disease, *UCD* Unconfirmed Celiac Disease, *PCD* Potential Celiac Disease, *tTg* tissue transglutaminase type 2 antibodies, *EMA* endomysial antibodies, *ULN* Upper Limit of Normal

^a^at least tTG or EMA positive or both positive; ^b^EMA or HLA positive but not both done. This category was not taken into account for comparison of the variables considered in the study

Crosstabs and stepwise statistics were generated by SPSS and t-Test, Relative Risk (RR) and Positive Predictive Value (PPV) were estimated for each variable assuming histology as the gold standard.

## Results

### Population

Demographic data of all the patients, and according to the final diagnosis, are shown in Table 3.

**Table 3:** Demographic data according to the final diagnosis

	No. (%)	Gender (F/M)	Age (M ± SD)
PATIENTS	1974	1109/865	4.83 ± 4.72
Confirmed	511 (25.9%)	320/191	4.1 ± 4.11
Unconfirmed	1391 (70.5%)	739/652	5.11 ± 4.89
Potential	42 (2.1%)	28/14	4.25 ± 4.51
Diagnosed according new ESPGHAN guidelines	14 (0.7%)	10/4	3.97 ± 3.59
High probability	16 (0.8%)	12/4	2.98 ± 2.93

**Fig. 1:**
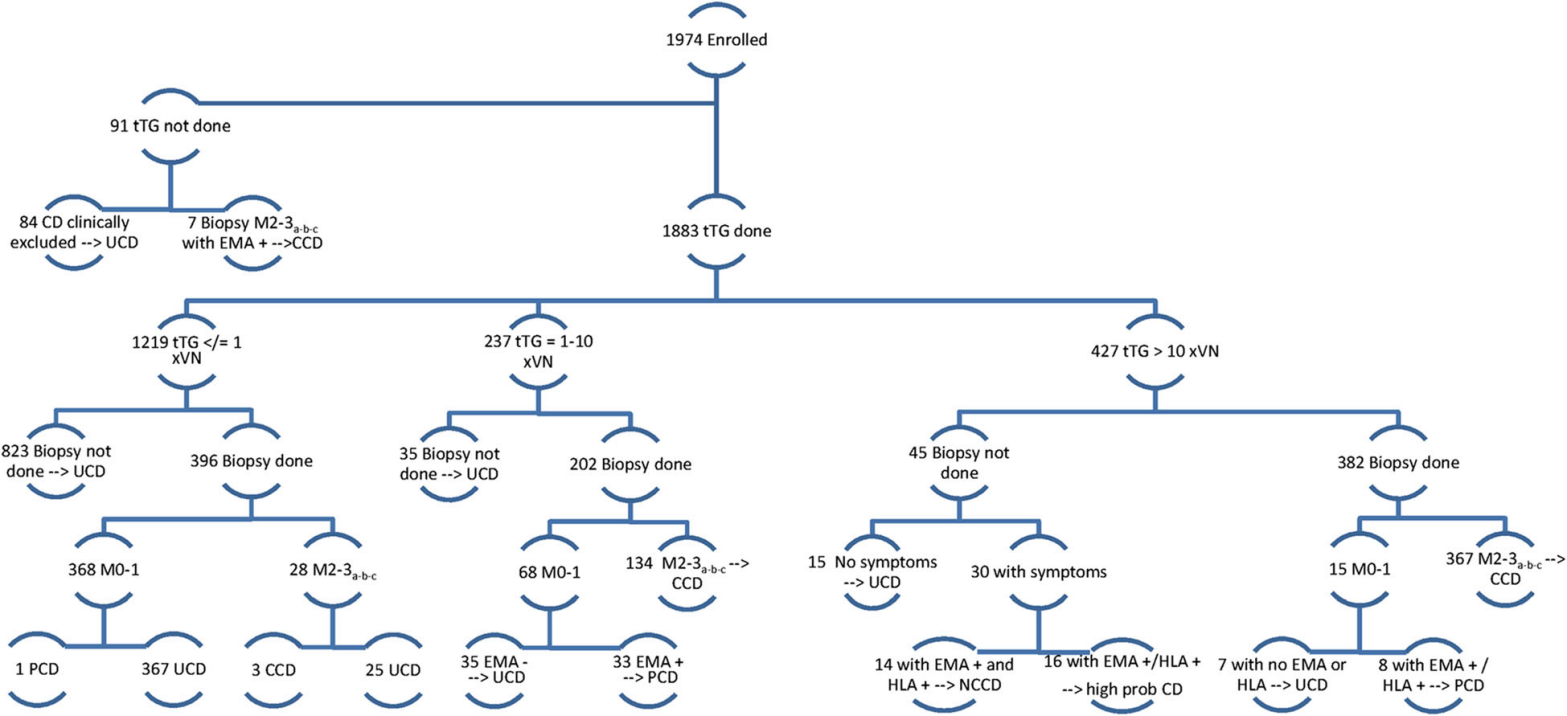
Prospective study design. Detailed legend: tTG: tissue transglutaminase; CD: celiac disease; CCD: confirmed celiac disease; UCD: unconfirmed celiac disease; PCD: potential celiac disease; NCCD: celiac disease diagnosed according to new EPSGHAN criteria; M: Marsh degree

There were 1974 patients enrolled in the study, mean age 4 years, 10 months; 865 male, 1109 female. A global view of the classification and investigations performed by each Centre can be seen in the Flow Chart (Fig. 1) and in Table 4. CD was confirmed (CCD) in 511 (25.9%) and was unconfirmed (UCD) in 1391 (70.5%) patients. Apart from 14 patients diagnosed as having CD according to the new ESPGHAN guidelines (NCCD: New Criteria Celiac Disease), 2.47% of the final amount of diagnoses of CD, 43 patients were classified as having Potential Celiac Disease (PCD), 7.4% of the CD population, and 16 defined as having “high probability” of CD, as EMA or HLA were positive, but not both were done.

**Table 4:** Distribution of patients by study centers and according to the final diagnosis and performed diagnostic investigations

Country	N. Pts.	N. CCD + NCCD^a^	N. UCD	N. PCD	N. tTG	N. EMA (in CCD)	N. Intestinal biopsy (in CCD)	N. HLA
Albania	45	25	20	0	45	7 (1)	32 (25)	0
Algeria	71	17	54	0	70	6 (2)	54 (17)	0
Croatia	310	12 + 1	293	3	304	25 (6)	202 (12)	14
Egypt	200	5	195	0	159	0	21 (5)	0
Greece	46	35	10	1	46	40 (31)	46 (35)	27
Italy (ME)	332	111	199	15	307	273 (108)	142 (111)	13
Italy (NA)	339	152 + 9	153	17	330	281 (151)	202 (152)	109
Malta	27	14	13	0	26	16 (14)	27 (14)	0
Montenegro	18	11	4	2	17	5 (3)	16 (11)	11
Morocco	106	45	61	0	100	1 (1)	90 (45)	0
Slovenia	234	29 + 1	203	1	234	234 (29)	34 (29)	66
Spain	40	4 + 3	32	1	40	10 (4)	8 (4)	9
Tunisia	67	22	44	1	66	23 (11)	43 (22)	0
Turkey	139	29	109	1	139	5 (1)	85 (29)	133
TOTAL	1974	511 + 14	1391	42	1883	926 (362)	1002 (511)	382

*N. Pts* Number of Patients, *N. CCD* Number of Patients with Confirmed Celiac Disease, *N. UCD* Number of Patients with Unconfirmed Celiac Disease, *N. PCD* N. of Patients with potential celiac disease, *N. tTG* Number of tissue TransGlutaminase antibody assays, *N. EMA* Number of EndoMysial Antibody assays

^a^diagnosed according to new ESPGHAN criteria omitting biopsy

### Symptoms

The proportion of asymptomatic cases enrolled for familiarity and of symptoms in CCD and UCD are shown in Table 5. A higher prevalence of asymptomatic cases, food refusal, globose abdomen and paleness was found in CCD, whereas abdominal pain and constipation were more common in the UCD. No difference was found for diarrhoea and failure to thrive between the two groups.

**Table 5:** Distribution of symptoms in UCD and CCD patients

Symptoms	UCD (%)	CCD (%)	χ^2^	*p*
No symptoms	93 (6.7%)	120 (20.6%)	82.4	<0.0001
Abdominal pain	402 (28.9%)	69 (11.8%)	65.9	<0.0001
Constipation	89 (6.4%)	17 (2.9%)	9.8	0.002
Diarrhea	297 (21.4%)	145 (24.9%)	2.93	0.087
Failure to thrive	266 (19.1%)	98 (16.8%)	1.46	0.227
Food refusal	16 (1.2%)	14 (2.4%)	4.3	0.038
Globose abdomen	29 (2.1%)	22 (3.8%)	4.66	0.031
Mood changes	5 (0.4%)	6 (1%)	3.33	0.068
Paleness	46 (3.3%)	31 (5.3%)	4.43	0.035
Vomiting	103 (7.4%)	30 (5.1%)	3.34	0.068
Others	45 (3.2%)	31 (5.3%)	4.81	0.028

*UCD* Unconfirmed Celiac Disease, *CCD* Confirmed Celiac Disease

### Diagnostic tools

Table 4 shows the frequency of various investigations performed in different countries: in 5 countries, HLA, and in one EMA assays were not available. HLA was not performed in Malta because it was not considered necessary.

### Serology

In 91 patients, tTG antibodies were not performed; out of these, 7 patients showed positivity for EMA and histology, allowing to classify them as CCD, while in the other 84 cases CD was excluded (thus, they were classified as UCD, see Fig. 1). Of 1883 patients evaluated for tTG: 1219 were negative, while 664 were positive.

Among the 1307 UCD patients in whom tTG were performed, 1215 resulted negative, while 92 resulted positive: 70/1307 (5.4%) had tTG titre higher than 2 x ULN, and 22/1307 (1.7%) had tTG titre higher than 10 x ULN.

Four patients received diagnosis of CD despite having negative titre of tTG in presence of EMA positve: one was classified as PCD because of Marsh type 0, while the other 3 presented a Marsh type equal to or higher than 2. tTG sensitivity and specificity were 99.3% (95% CI 98.6-99.9%) and 93% (95% CI 91.6-94.3%) respectively, with a RR 1888.53 (95% CI 690.61-5164.35) of being celiac.

**Fig. 2:**
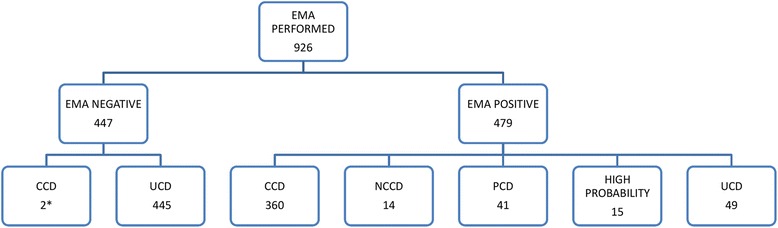
Role of EMA assay in the diagnosis. Detailed legend: *tTg and Histology positive

Results of EMA assay are shown in Fig. 2. EMA sensitivity and specificity were 99.5% (95% CI 98.8-100%) and 90.1% (95% CI 87.5-92.7%) respectively.

Both EMA and tTG were performed in 464 patients who underwent intestinal biopsy as well: 66 were EMA negative and 398 EMA positive. The reliability of tTG versus EMA is defined by its sensitivity 95.7% (95% CI 93.86-97.54%) and specificity 95.8% (95% CI 93.91-97.69%). High titers of tTG > 10 x ULN were found in 427 patients, out of whom EMA were performed in 289 and were found absent in only 3 patients (1.04%). Intestinal biopsy was performed in 2 of these latter patients and in both, histology showed a Marsh type 3c confirming CD diagnosis. The third was classified as UCD because of having done neither HLA nor intestinal biopsy.

In 135 patients with tTG > 10 x ULN, HLA genotyping was performed and in all of them it was compatible with CD.

Out of the 413 patients with tTG > 10 x ULN (excluding 14 patients diagnosed according to the new ESPGHAN criteria) both EMA and HLA were performed in 88. In all these patients a duodenal biopsy was performed, and in 85/88 the diagnosis would have been correctly made according to the new criteria; in 3 (3.41%) the diagnosis of PCD would have been missed in absence of the biopsy.

Biopsy was performed in 382 out of the 427 patients with high titre tTG, and histology showed at least a Marsh type 2 in 367 patients (96.1%). Thus, in this subgroup of patients, the positive predictive value of tTG > 10 x ULN was 96.1% (95% CI 94-97.9%).

### Biopsy

Of 1974 cases included in this study, 1002 underwent an intestinal biopsy and in 542 at least Marsh type 2 mucosal damage was found: 511 (94%) were classified as CCD, because of positivity of tTG or EMA, 31 (6%) were classified as UCD because of negativity of both tTG and EMA.

Out of 460 patients with Marsh type 0-1, 42 (9%) were classified as PCD in light of the positivity of EMA or tTG antibodies. In particular, 8 of them had tTG > 10 x ULN, 6 between 5 and 10 x ULN, 27 between 1 and 5 x ULN. One patient had a negative titre of tTG (0.9 x ULN), but he was symptomatic (abdominal pain) and had positive EMA with compatible HLA.

In 364/460 with a Marsh type 0-1 at biopsy, no antibodies were produced and they were classified as UCD.

Overall, 568 patients were diagnosed as having CD (including CCD, NCCD and PCD), 2.47% of whom according to the new guidelines.

**Fig. 3:**
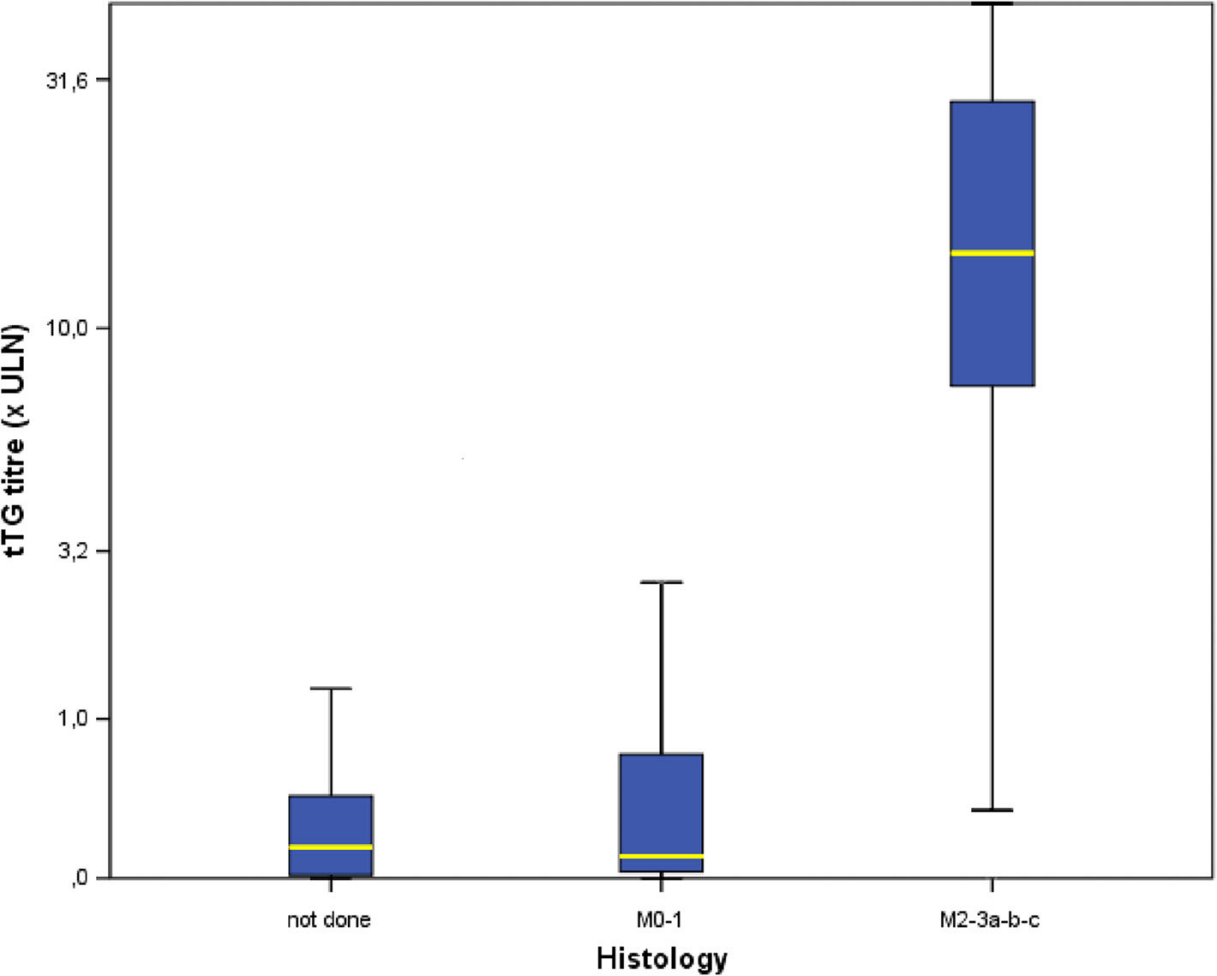
Correlation among tTG titre and villous atrophy

The presence of high titre of tTG correlates with villous atrophy (Fig. 3).

### HLA

Less than 20% (382) of the total number of enrolled patients underwent a HLA analysis: in 63, that were both DQ2 and DQ8 negative, CD was finally excluded they were classified as UCD.

### Associated diseases

About 80% of the total study population had no other disease. The significant differences in prevalence of associated diseases in UCD and CCD are shown in Table 6. The frequency of thyroiditis, type 1 diabetes mellitus and dermatitis herpetiformis was higher in CCD, whereas IgA deficiency and hypertransaminasemia were higher in UCD.

**Table 6:** Significant differences in prevalence of associated diseases in unconfirmed celiac disease (UCD) and confirmed celiac disease (CCD)

Associated diseases	UCD (%)	CCD (%)	χ^2^	*p*
Thyroiditis	1	5.7	38.7	0.0001
Type 1 Diabetes Mellitus	7	3	16	0.0001
Dermatitis herpetiformis	0.1	1.5	17.1	0.0001
IgA deficiency	1.8	0.3	6.3	0.012
Hypertransaminasemia	2.7	0.7	7.87	0.005

## Discussion

Our study, for the first time, provides a wide picture of CD diagnosis feasibility in 13 Mediterranean countries with different health resources and facilities. It is also the largest one investigating antibody diagnostics in paediatric CD that prospectively evaluates the new ESPGHAN guidelines in different countries including those with poor resources.

In all participating countries, the diagnosis of CD relied on histology of duodenal biopsy, but other diagnostic procedures were not always available. In particular, in 5 countries, HLA, and in 1, EMA were not performed. Even though HLA was not required when the diagnosis was not in doubt, in some countries the limitation of performing it derives from its cost. The same is true for EMA assay.

On the other hand, in order to apply what is suggested by the new ESPGHAN guidelines, apart from the presence of symptoms and high antibody levels, HLA has to be compatible and EMA have to be present if the diagnosis of CD is to be made without a biopsy. In 4 centres, the diagnosis of CD was made for a total of 14 patients according to these new guidelines, omitting a duodenal biopsy.

Even though CD can be reliably diagnosed following the latest ESPGHAN and BSPGHAN guidelines [16], as not all diagnostic procedures required by the new guidelines are available in all countries, our results may suggest to further simplify the new ESPGHAN guidelines. As a matter of fact, it is not useful nor necessary to perform an expensive determination, such as HLA, as in all patients with tTG higher than 10 x ULN who had HLA heterodimer determined it was compatible with CD. Moreover EMA, which is not easily performed in all countries, does not add diagnostic accuracy to tTG, as suggested by the cases in which both tests were performed and tTG was > 10 x ULN. In 3 cases of our study, in whom EMA were negative, histology showed a picture that allowed us to make the diagnosis of CD. Rather than determining EMA, in the presence of such a high titre tTG, it is better to repeat a second test for tTG according also to what is suggested by the Joint BSPGHAN and Coeliac UK guidelines for diagnosis and management of CD in children [17]. In a commentary regarding applicability of the new ESPGHAN guidelines for diagnosing celiac disease in children from resource limited countries [14], it has been suggested that positive HLA-DQ2/DQ8 serotype and EMA are necessary in order to apply the ESPGHAN guidelines for serological diagnosis of CD, and that CD should not be diagnosed on the basis of a single high tTG-titre. Our study comprising many countries with limited resources suggests that both HLA and EMA may be omitted and CD may be diagnosed on the basis of repeated high tTG titres. Altogether, the diagnosis would have been correctly made according to the new criteria in 85 out 88 patients with tTG > 10 x ULN who underwent tTG and EMA determination, together with intestinal biopsy. In 3 patients (3.41%), the diagnosis of PCD would have been missed in absence of the biopsy.

Thus, some practical issues need to be addressed. In adopting the new ESPGHAN criteria, it has to be kept in mind that there is a chance that patients might have a potential CD. In a prospective cohort study [18], which describes the long term natural history of potential CD by a 9 years follow-up, the risk of becoming atrophic was estimated at 18% especially in subjects with persistent positive serology, while serology became negative in 20% of potential patients on follow up. Even if rarely, potential celiac patients showed a TTG value >10 x ULN: hence this limit does not exclude the chance of finding potential celiac cases. Starting from this point, the decision to omit biopsy and the chance of being a potential CD should be thoroughly discussed with families considering the life-long diagnosis of CD. The new ESPGHAN guidelines also state that it is important to be precise in the clinical evaluation of patients and to perform prospective research studies. To the best of our knowledge, our prospective study is the largest one including 511 (+14) CD patients and 1391 controls and it shows that symptoms are not able to assign a pre-test probability to serological tests. On the other hand, Webb et al. [11] found no difference in terms of diagnostic accuracy of tTG in asymptomatic children diagnosed as having CD detected by screening.

We are aware of the limitations of our study, mostly due to the unequal distribution of enrolled patients across participating countries, although the total number is the largest one reported.

Therefore, further prospective multicentre studies with an homogeneous enrolment of patients should be planned also in order to try a further simplification of ESPGHAN guidelines.

Moreover, due to different test kinetics the 10 x UNL is not the same with all tTG tests. However, all the centres participating in the study utilized one of the 14 most frequently applied serum tTG assays in Europe taken in consideration in the new ESPGHAN guidelines Appendix. All these assays underwent United Kingdom National External Quality Assessment according to which the 10 x ULN was suggested by the new guidelines in order to omit intestinal biopsy. Considering that all the centres took at least 4 duodenal biopsy samples always including a sample from the bulbus we are confident that histology is the right gold standard chosen in this study.

## Conclusions

This is the largest prospective study providing a wide picture of CD diagnosis feasibility in Mediterranean countries with different health resources and facilities. The results of our study show that CD diagnosis still relies on intestinal biopsy in the Mediterranean area. New ESPGHAN criteria are not applicable in 5 countries due to lack of resources needed to perform HLA genotyping and, in one country, EMA assay. Further simplification of the new ESPGHAN guidelines might be made according to what preliminarily the present results suggest if confirmed by new prospective studies. This would be a result of great value especially for countries with limited resources, even though the chance of a potential CD has to be taken into account and discussed with the families when intestinal biopsy is omitted.

## Abbreviations

BSPGHAN: British society for pediatric gastroenterology, hepatology and nutrition
CCD: Confirmed celiac disease
CD: Celiac disease
ESPGHAN: European society for pediatric gastroenterology, hepatology and nutrition
NCCD: New criteria celiac disease
PCD: Potential celiac disease
PPV: Positive predictive value
RR: Relative risk
tTG: tissue transglutaminase type 2 antibodies
UCD: Unconfirmed celiac disease
ULN: Upper limit of normal
